# Editorial Announcements

**Published:** 1913-09

**Authors:** 


### Editorial Announcements

Growth of the Journal. Two years ago the regular issue of the Journal consisted of 92 pages, including covers. During the past year the total has been 100 pages. Beginning with the present issue the total will be further increased to 108 pages, representing an increase of a trifle over one-sixth in two years. The circulation has increased a little over 50 per cent. in two years. The loyalty of our subscribers is shown most convincingly in the fact that the most considerable item in the loss of subscriptions has been on account of death. As many readers have asked how they can assist the Journal, we mention the following needs: 1. Prompt and brief communication of news items, personals, obituaries, etc., especially outside of Buffalo; 2. Assistance in reviewing the great mass of current literature received, especially American monthlies in lines outside the editor's immediate professional interests and knowledge, and journals in

Japanese, Italian, Swedish and Greek. For obvious practical reasons, assistance of this nature is almost confined to residents of Buffalo and it requires fairly regular attention. 3. Missionary work to increase our subscriptions and advertising. A good word in passing is all that is asked in these matters.

POSTAGE STAMPS. We receive a good many foreign postage stamps. Any physician is welcome to these if he or any member of his family is interested in philately.

BILLS FOR SUBSCRIPTIONS were inclosed in the August issue. Please look for yours if you have not already found it and give it prompt attention, especially if there is any error. There are three reasons for asking for promptness. The first is too obvious to mention. The second is that the standing of a periodical with the Postoffice depends upon its paid-up subscription list. The third is the enormous saving of time when anything can be dismissed from attention as finished.

ADDITION TO STAFF OF JOURNAL. Dr. H. I. Davenport of Auburn, a graduate of Johns Hopkins of the class of 1905, has accepted an invitation to join the staff of Associate Editors. Pending the appointment of a representative for Syracuse, he will, so far as possible, supply us with items from that city as well as the immediate vicinity of Auburn. Dr. Davenport is especially interested in bacteriology and pathology and has a laboratory for Chemic and Microscopic Diagnosis. We take great pleasure in giving him a hearty welcome.

THE DEAD CENTER. We try to be fairly up-to-date in all departments of the JOURNAL, especially Society Meetings, Topics of Public Interest, Personals and Obituaries. Unfortunately, we pass a dead center between the twentieth and the last of each month while the JOURNAL is being printed. A very brief and important notice can usually be inserted by displacing other matter in type, up to the twenty-fifth. Otherwise, copy received after the twentieth of one month cannot appear until the issue of the second month following.

